# The Rational Treatment of the Constitutional Factor in the Causation of Hay-Fever

**Published:** 1897-11

**Authors:** Charles Prevost Grayson

**Affiliations:** Lecturer on laryngology and rhinology in the Medical Department of the University of Pennsylvania; Chief of Department for Diseases of Throat and Nose, University Hospital


### Selections.

### THE RATIONAL TREATMENT OF THE CONSTITUTIONAL FACTOR IN THE CAUSATION OF HAY-FEVER.

By CHARLES PREVOST GRAYSON, M. D.,
Lecturer on laryngology and rhinology in the Medical Department of the University of Pennsylvania; Chief of Department for Diseases of Throat and Nose, University Hospital.

So LONG as almost all the laity and a large proportion of medical men continue to regard hay-fever as incurable; so long as a number of general practitioners persist in the attempt to cure it solely through the agency of drugs, and a number of rhinolaryngologists seek to attain the same and solely through intranasal medication or surgery; so long, in fine, as the ultimate nature of the disease is imperfectly understood or even regarded with indifference, so long will there be ample justification for brief papers that shall not only insist that the disease can almost invariably be cured, but shall show how this may be accomplished.

A moment's reference to the several trivial theories that attempt to explain the evolution of the disease cannot be avoided; but ferments, microörganisms, acid nasal secretions and the like are all idle speculations that add nothing whatever of value to our means of treating hay-fever. They merely hang upon the skirts of what we positively know of the malady and serve only to distract attention from the broad therapeutic principles that must underlie its successful management. What we do know of it can be epitomised in a few words. Three factors compose the causative combination ; of these, two—the existence of an external irritant and of some intranasal abnormality—are accepted without discussion. As to the third, however, there is considerable diversity of opinion. That it is constitutional or diathetic is generally believed, but whether it is sufficiently well defined to be given a specific name, such as " the neurotic temperament " or the " gouty or uric acid diathesis," or some other equally distinctive title, is still far from being settled. The very fact that opinions concerning it differ so widely, as the two suggested titles indicate, is evidence of the want of unanimity concerning its precise nature. As a matter of fact, however, names in a case of this kind are merely ornamental, and are, indeed, objectionable, for the reason that they tend to discourage separate and searching study of each case. Even if we grant that a certain number of hay-fever sufferers are unquestionably people of " neurotic temperament," while others, without being at all neurotic, are of a certainty gouty, can we not profitably look beneath these titles and recognise the fact that they designate dyscrasia, which are merely different offshoots from a parent weed that is rooted in defective nutrition ? Is it not, therefore, to this primary departure from normal processes that we should address our corrective measures ? Of what use is it to administer to the neurotic individual the most potent of nervines, or to the gouty patient the best of antilithics, if underlying both of these conditions there is a cause upon which none of these remedies exerts a noticeable influence ?

There is no need to analyse the expression "defective nutrition" further than to state that it is meant to include all the phenomena of metabolism—constructive, destructive and eliminative. Disturbance of one of these means disturbance of all, and, as a final observation upon this subject, we may note the fact that with continued absorption of toxic materials from the intestinal tube, or with persistent incomplete elimination of the products of sub-oxidation, it is only a question of time when autotoxemia will provide us with any of the functional neuroses from hay-fever and asthma to chorea and epilepsy.

With this broad conception of the constitutional factor that shares in the genesis of hay-fever, its rational treatment will evidently demand far more than a mere juggle with drugs, be this ever so skilful. The end to be attained is the removal and checking of further production of every toxic body that can so enfeeble the nerve centers as to render possible this annual vasomotor paresis that affects the intranasal circulation. The merely eliminative portion of this procedure is not a matter of great difficulty, but when we grapple with the problem of preventing further contamination of the blood current we find that it is no trifling task that we have undertaken. And this is so because our success so largely depends upon the intelligence of the patient and the fidelity with which he coöperates with us. To secure this coöperation, and retain it until the victory is gained, requires both tact and firmness. A sensible patient will not regard it as an evidence of weakness if we confess at the outset our inability to conquer the disease single-handed; and if he be well impressed with a sense of his own responsibility in the winning of the battle, he will seldom have to be coaxed or threatened in order that he shall do his share. I regard it as a means of saving time, temper and reputation to have a clear understanding with the patient, before the first blow is struck, as to what we will expect of him. The very essential restoration of the nose to a state of health is exclusively our own affair, and we can effect that unaided; but when it comes to restoring the patient's nutritive processes to functional perfection, to rehabilitating his more or less dilapitated nervous system, he must join with us in the struggle that it involves. The main battle-ground is to be his own gastro-intestinal tract, and, as he is to be the one sentinel throughout the conflict, much depends upon his constant vigilance and his strict obedience to orders.

One more word regarding the character of our enemy and we will abandon this figurative strain and descend to formal English. It is a host of hygienic blunders and, perhaps, wilful bad habits that we are to meet and overcome—self-indulgence (a term covering a multitude of sins), irregularities connected with the patient's hours for meals, for work, rest and play, indiscretions of diet, lack of exercise, objectionable fancies in matters of clothing and bathing, and, finally, vicious excesses—alcoholic, narcotic or sexual. All these make quite a formidable array and they are to be subdued not by any single brilliant bit of strategy, but only by a prolonged and unrelenting struggle that requires immense patience and determination. In considering the ways and means, I make it a rule in my lectures at the University of Pennsylvania to enter quite thoroughly into details,[1] but in these columns this is uncalled for, and I have only to emphasise certain points that I regard of special interest and importance.

1. University Medical Magazine, July, 1896.

The accurate adaptation of the diet to any inherited or acquired morbid state that the patient presents is one of the first essentials. It is not enough merely to reduce the starches and sweets if he be neurotic, or the nitrogenous foods if he be gouty—a shallow routinism would dictate that much; but the age, the sex, the occupation, the whole environment of the patient must be separately studied and provided for in the dietary scheme.

In the closest possible association with the regulation of food is that of exercise. By the majority of physicians scarcely anything is prescribed with less intelligence, with less apprehension of its possibilities for good or evil, than this agent. Judiciously advised, with careful reference to the condition and needs of the patient, it is worth all the drugs in the pharmacopeia. Iron is, indeed, a veritable specific for these cases of nervous and muscular asthenia, but it should be prescribed in the form of dumb-bells. Is it not an egregious folly to prefer the temporary and deceptive benefits of strychnine to the permanent and real ones that attend the proper use of the muscles? Is the passive cutaneous leakage that transpires in the hot-air chamber of a Turkish bath establishment to be compared in therapeutic value to the natural and healthful stimulation of the sweat glands that accompanies active exercise? I believe in nothing more firmly than that if a man takes care of his muscles his nerves will take care of themselves. And yet there is no closing our eyes to the fact that, to the average man, exercise is distasteful, is hard work, yea, even drudgery. It requires all the eloquence we possess to convince him, first, of its necessity, and then to persuade him to adopt and adhere to it. We are not, therefore, to be content with lightly telling him to "take a little more exercise," to walk where now he rides, or, in these days, to buy himself a wheel—and then to leave the manner of its use to his own unenlightened discretion. The more stress we lay on this portion of the treatment, the more explicit we are in our instructions concerning it, the more will the patient be impressed with its importance and the necessity for doing what he is told. In brief, then, it is in the skilful blending of diet and exercise, and their application in proper proportions to each of these patients, that we have the one rational and reliable method of removing the constitutional factor that is so active in the causation of hay fever. Within a reasonable time the digestive tract is restored to a state of sanitary purity, general nutrition is established upon a firm foundation, and the previously unstable nervous system, steadied and invigorated, is enabled to resist such disturbing influences as once proceeded from the contact of atmospheric irritants with the hyperesthetic pituitary membrane; the vasomotor centers resume their domination over the nasal erectile structures, and the distressing phenomena of hay-fever are known no more.

Of course, that which I have written here concerning diet and exercise applies with equal force to both sexes and all ages. Certain modifications, it goes without saying, are to be made with respect to differences in sex, age and occupation; but the essential idea is to put the patient upon a course of strict training that will bring out all his capacity for self-denial and self-help, that will effect a most salutary change in his whole *morale*, and that will so completely remove the hyperesthesia of the nasal sensory nerves as to make them contemptuously indifferent to, even unconscious of, a species of irritant that once sufficed to throw them into a state of violent commotion. There is nothing brilliant, it is to be confessed, about this method of removing the constitutional factor, but for what it lacks in brilliancy it more than makes amends in certainty. It is, often enough, slow and tedious, but to patients possessed of grit and determination it brings a sure reward. It penetrates to and combats the very beginnings of pathogenesis, and with this fact in mind we can scarcely be over-confident in anticipating and prognosticating success.—*Therapeutic Gazette.*

---

Promiscuous Use of Handkerchiefs.—One of the commonest maladies is "cold in the head," or, as it is technically called, "coryza." It is notoriously infectious and the means of communication is the discharge from the nostrils. I am satisfied, from repeated observation, that this troublesome affection often spreads through a family of children and then through an entire household through the promiscuous use of the pocket handkerchief. A little child comes to the nurse with the request, "blow my nose." This is carelessly or thoughtlessly done with the parent's or attendant's pocket handkerchief, which has become infected and spreads the attack. In other cases the soiled pocket handkerchief is allowed to dry without disinfection and the dried discharge from the diseased mucous membrane of the nose is then diffused through the air, spreading the malady just as measles is spread.—Moore, in Proceedings of Dublin Sanitary Association.—*Med. Age.*

